# Distinct patterns of soluble leukocyte activation markers are associated with etiology and outcomes in precapillary pulmonary hypertension

**DOI:** 10.1038/s41598-020-75654-w

**Published:** 2020-10-29

**Authors:** Tove Lekva, Lars Gullestad, Kaspar Broch, Pål Aukrust, Arne K. Andreassen, Thor Ueland

**Affiliations:** 1 Research Institute of Internal Medicine, Oslo University Hospital, Rikshospitalet, Sognsvannsveien 20, 0027 Oslo, Norway; 2 Department of Cardiology, Oslo University Hospital, Rikshospitalet, Oslo, Norway; 3 Faculty of Medicine, University of Oslo, Oslo, Norway; 4 Center for Heart Failure Research, K.G. Jebsen Cardiac Research Center and Center for Heart Failure, University of Oslo, Oslo, Norway; 5 Section of Clinical Immunology and Infectious Diseases, Oslo University Hospital, Rikshospitalet, Oslo, Norway; 6 K.G. Jebsen Thrombosis Research and Expertise Center, University of Tromsø, Tromsö, Norway

**Keywords:** Biomarkers, Cardiology

## Abstract

Activation of inflammatory processes has been identified as a major driver of pulmonary vascular remodeling that contributes to the development of precapillary pulmonary hypertension (PH). We hypothesized that circulating markers of leukocyte activation, reflecting monocytes/macrophages (sCD163, sCD14), T-cells (sCD25) and neutrophils (myeloperoxidase [MPO], neutrophil gelatinase-associated lipocalin [NGAL]) activity, could give prognostic information in precapillary PH. Circulating markers of leucocyte activation, sCD163, sCD14, sCD25, MPO and NGAL were measured by enzyme immunoassays in plasma from patients with idiopathic PAH (IPAH; n = 30); patients with PAH related to associated conditions (APAH; n = 44) and patients with chronic thromboembolic PH (CTEPH) (n = 32), and compared with 23 healthy controls. Markers of leucocyte activation were elevated in precapillary PH with particularly high levels in APAH. The elevated levels of monocyte/macrophage marker sCD163 was independently associated with poor long-term prognosis in the group as a whole, and elevated levels of sCD25 was associated with poor prognosis in APAH, while elevated levels of sCD163 and NGAL was associated with poor prognosis in IPAH and CTEPH. Our data show leucocyte activation in precapillary PH with different profiles and impact on prognosis according to etiology. The association of sCD163 with poor outcome in fully adjusted model may be of particular interest.

## Introduction

In precapillary pulmonary hypertension (PH) pulmonary vascular remodeling leads to high pulmonary arterial pressure, right ventricular failure and death^1^. PH may develop in patients with systemic autoimmune and infectious disorders, in chronic thromboembolic pulmonary disease (CTEPH), or in familial or idiopathic pulmonary arterial hypertension (IPAH).

In recent years, inflammation has been identified as a major driver of the pulmonary vascular remodeling contributing to the development of precapillary PH^2,3^. Inflammatory cells such as T-cells and monocytes interact with pulmonary vascular cells and macrophages to generate vascular remodeling in the lungs of PH patients^4^. The inflammatory mediators that are released from these activated immune cells are elevated in plasma/serum in PH and correlate to the disease severity and progression^5,6^. However, cytokines and chemokines frequently circulate at low levels, just above the detection limit of the actual assay, which limit their analytical performance and discriminatory properties beyond established clinical markers. On the other hand, certain markers reflecting activation of various leucocyte subsets such as monocytes, T-cells and neutrophils, circulate at readily detectable levels, but have not been evaluated in PH, with a few exceptions^7,8^.

We hypothesize that circulating markers of leukocyte activation, reflecting monocytes/macrophages (soluble [s] CD163^9^, sCD14^10^), T-cells (soluble interleukin 2 receptor [sIL-2R]/CD25^11^ and neutrophils (myeloperoxidase (MPO)^12^, neutrophil gelatinase-associated lipocalin (NGAL)^13^) activity would be elevated in patients with precapillary PH. We assumed that these markers would reflect vascular resistance and cardiac function, and could give information on long-term prognosis in these patients.

## Methods

### Study population

We studied 106 patients with precapillary PH in New York Heart Association (NYHA) functional classes II–IV (Table 1). Precapillary PH was defined in accordance with international guidelines as a mean pulmonary artery pressure (MPAP) ≥ 25 mm Hg at rest, a pulmonary capillary wedge pressure (PCWP) ≤ 15 mmHg and a PVR > 3.0 Wood units (WU)^14^. Based on the same guidelines, the study population was divided into three clinical subgroups: (1) patients with idiopathic PAH (IPAH; n = 30); (2) patients with PAH related to associated conditions (APAH; n = 44) [connective tissue disease, n = 21; HIV infection, n = 3; portal hypertension, n = 7; congenital heart disease, n = 13] and (3) patients with CTEPH (n = 32). Occlusive thromboembolic disease was verified with pulmonary angiograms. For comparison, we studied 23 healthy controls with CRP levels within normal limits. The investigation conforms to the principles outlined in the Declaration of Helsinki. The Regional Committee for Medical Research Ethics of Southern Norway approved the study, and informed consent was obtained from each subject.

**Table 1:** Characteristics of the study group.

	IPAH (n = 30)	APAH (n = 44)	CTEPH (n = 32)	*p*-value
Age, year	43.1 (14.9)	44.2 (13.7)	56.7 (12.6)*†	< 0.001
Sex, male	23% (7)	27% (12)	50% (16)*†	0.047
RAP, mm Hg	6.8 (4.2)	6.0 (4.6)	5.8 (3.6)	0.672
MPAP, mm Hg	55 (13)	51 (16)	42.7 (10.6)*†	0.001
PCWP, mmHg	6.5 (3.7)	6.1 (3.0)	7.0 (3.1)	0.483
Cardiac index, L/min/m^2^	1.8 (0.4)	2.2 (0.6)*	2.2 (0.7)*	0.002
PaSO2, %	58 (8)	59 (11)	62 (9)	0.204
FaSO2, %	94.3 (3.1)	90.9 (6.5)*	91.0 (4.5)	0.007
PVR, wood units	15.9 (5.5)	13.1 (6.3)*	9.3 (4.4)*†	< 0.001
Peak VO2, ml/kg/min	12.0 (4.2)	12.9 (5.3)	13.3 (4.8)	0.515
NT-proBNP, pmol/L	341 (266)	297 (312)	185 (175)*	0.049
Creatinine (µmol/L)	95 (40)	85 (27)	90 (16)	0.085
eGFR (ml/min/1.73 m^2^)	71.7 (24.3)	80.8 (25.0)	72.9 (13.0)	0.224
CRP (mg/L)	4.7 (2.4, 6.6)	5.0 (2.8, 7.0)	3.4 (2.0, 5.5)	0.106
SpD (ng(/mL)	16.7 (8.1, 27.4)	24.4 (11.5, 48.3)*	29.8 (14.4, 36.0)*	0.038
PDE-5 inhibitor	50% (15)	50% (22)	0 (0)*†	< 0.001
Prostaglandin I2 (PGI_2_)	47% (14)	16% (7)*	3% (1)*	< 0.001
Endothelin receptor antagonist	40% (12)	21% (9)	3% (1)*†	0.002

APAH associated pulmonary arterial hypertension; CTEPH, chronic thromboembolic pulmonary hypertension; FaSO_2_, Femoral artery oxygen saturation; IPAH, idiopathic pulmonary arterial hypertension; MPAP, mean pulmonary artery pressure; NT-proBNP, N-terminal pro-brain natriuretic peptide; PaSO_2_, Pulmonary artery oxygen saturation; PCWP, pulmonary capillary wedge pressure; RAP, right atrial pressure; PVR, pulmonary vascular resistance; eGFR, estimated glomerular filtration rate; SpD, surfactant protein D. Data are presented as mean (SD) for continuous data and as percentage for categorical data. The medications represent therapy at baseline at the time of blood sampling. The *p*-value to the right represents the test for trend determined by Kruskal Wallis (continuous data) or chi-square (categorical data).

p* < 0.05 versus IPAH, *p*† < 0.05 APAH versus CTEPH.

### Hemodynamics and cardiopulmonary exercise testing

Right-sided cardiac catheterization was performed with a thermodilution catheter inserted through the right jugular vein, with a catheter inserted into the right femoral artery for the monitoring of arterial blood pressure and blood gases. Hemodynamic measurements included heart rate and right atrial pressure (RAP), mean pulmonary artery pressure (MPAP), and pulmonary capillary wedge pressure (PCWP). Cardiac output was calculated as the mean of three thermodilution technique measurements. We calculated pulmonary vascular resistance (PVR) and cardiac index (CI). Oxygen saturation was measured in blood samples from the femoral (FaSO_2_) and pulmonary arteries (PaSO_2_). Within 24 to 48 h of right-sided cardiac catheterization, a symptom-limited exercise test was performed using a cycle ergometer (ER900; Jäger, Wurzburg, Germany) with a steady cadence of 60 rotations per minute. The work-load started at 20 Watts and was raised incrementally 5–10 W per minute. Peak oxygen uptake (peak VO_2_) was defined as the highest 30-s average of the oxygen uptake during the last minute of exercise.

### Blood sampling and biochemical analyses

Blood samples were collected from the pulmonary artery (PH patients), femoral artery (PH patients), and peripheral vein (healthy controls) in chilled, pyrogen-free EDTA tubes. Platelet-free plasma (centrifuged within 30 min at 2000*g* for 20 min) was stored in multiple aliquots at – 80 °C until analyses and thawed once only prior to biochemical analysis. sCD25, sCD14, sCD163, MPO, NGAL, CRP and Surfactant protein D (SpD) were analyzed in duplicate by enzyme immunoassays from R&D Systems (Minneapolis, MN, USA). For sCD14 measurements, 20% fetal calf serum (Thermo Fisher Scientific Inc, Waltham, MA, USA) were used in the sample diluent. N-terminal pro B-type natriuretic peptide (NT-proBNP) was determined by an electrochemiluminescence immunoassay on a Modular platform (Roche Diagnostics, Basel, Switzerland). All intra- and inter-assay coefficients of variation were < 10%.

### Statistical analysis

Differences between groups were tested using Mann–Whitney U test (2 groups) or Kruskal–Wallis test (> 2 groups). Differences in the distribution of categorical data were analyzed with chi-square test. Skewed markers of leucocyte activation were log transformed before analyses. Because the age and gender distribution differed between controls and patients, and across diagnostic categories of precapillary PH, group differences were compared by MANCOVA with age and sex as covariates and in some analysis also CRP and SpD. We performed Bonferroni-adjusted post-hoc tests. Changes in leucocyte markers over time were analyzed by the Wilcoxon paired test. Differences in changes in leucocyte markers between survivors and non-survivors were analyzed by comparing change values (Mann–Whitney U test). Associations between leucocyte markers and clinical and hemodynamic parameters were assessed by Spearman correlation.

The association between leucocyte markers and all-cause mortality were assessed by multivariable Cox proportional hazards models. Prior to analyses, the plasma concentrations of the leucocyte markers were normalized, i.e. ln-transformed and divided by standard deviations. For assessment of the prognostic value of baseline levels, two models, with incremental addition of co-variates, were used in addition to univariate analysis. Model 1 included age, sex, RAP, PaSO_2_, estimated glomerular filtration rate (eGFR), CI, CRP and NT-proBNP (as the strongest predictors in univariate analysis) and Model 2 added peak VO_2_. A propensity score was calculated based on these variables (without peakVO_2_). Propensity score adjustment was used when evaluating survival within etiologies (due to low numbers). Results are presented as adjusted hazards ratios (HRs) and 95% confidence intervals (CIs). Kaplan–Meier curves were constructed to visualize and evaluate (log rank test) differences in survival according to tertiles of leucocyte markers. Two-sided probability values were considered significant at *p* < 0.05.

## Results

Characteristics of the study population are shown in Table 1. Patients with CTEPH were older, more frequently male, had lower MPAP and PVR, and used less medication than patients with IPAH and APAH. The healthy controls (n = 23) were older (59 ± 8 vs. 48 ± 15, *p* < 0.001) than the patients, and fewer were males (28% vs. 65%, *p* = 0.016).

### Circulating leucocyte markers in plasma are elevated in precapillary pulmonary hypertension

**Figure 1:**
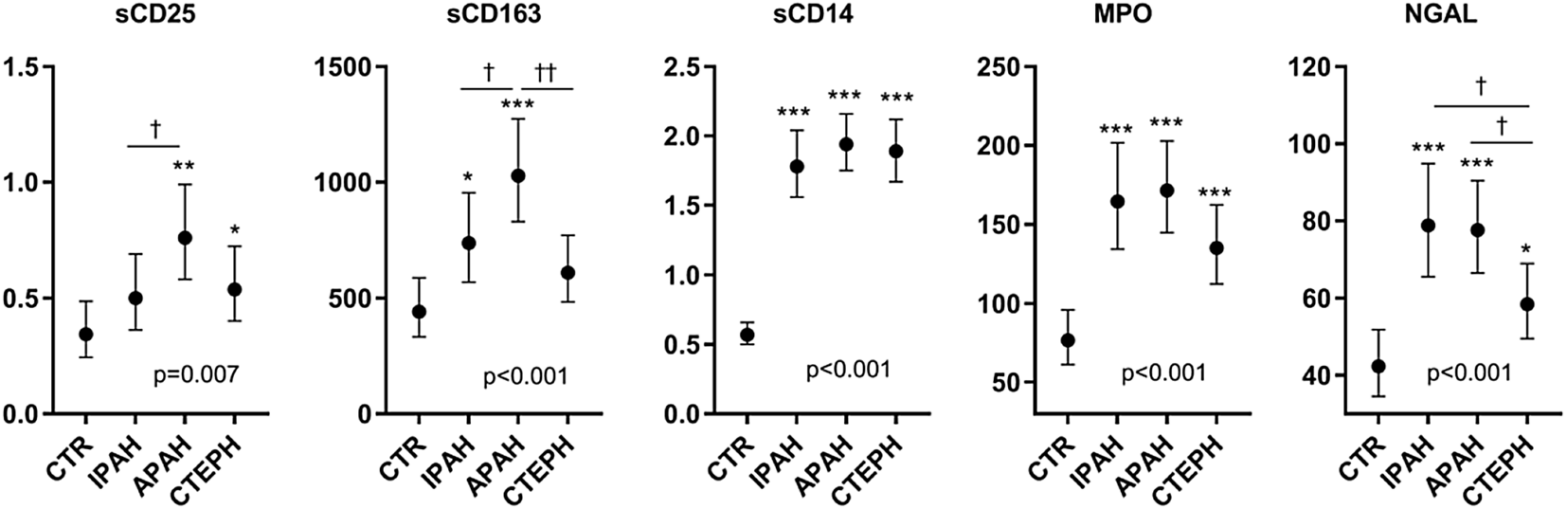
Levels of soluble markers of leukocyte activation in precapillary PH. Patients with PAH were classified as idiopathic PAH (IPAH, n = 30), associated PAH (APAH, n = 44), and chronic thromboembolic pulmonary hypertension (CTEPH, n = 32) and in healthy controls (CTR, n = 23). Data are given as estimated marginal means (ng/ml) adjusted for age and sex. *P* value represents the overall group effect. *p < 0.05, **p < 0. 01 ***p < 0.001 versus controls. †*p* < 0.05, ††*p* < 0.001 between etiologies.

Adjusted for age and sex, patients with APAH and CTEPH had elevated mixed venous blood (pulmonary artery) levels of sCD25 and patients with IPAH and APAH had elevated mixed venous blood levels of sCD163 compared with peripheral venous levels in controls (Fig. 1). All the diagnostic groups had higher age and sex adjusted levels of sCD14, MPO and NGAL compared to the healthy controls, although there were no statistical differences between the separate diagnostics PH groups. A similar pattern between patients and healthy controls was found when comparing samples from arterial blood (femoral artery, patients) and samples from peripheral venous blood in healthy controls (Supplementary Figure S2).

Significant differences across the diagnostic groups included higher levels of sCD25 and sCD163 in APAH compared to IPAH, and higher levels of NGAL in APAH and IPAH compared to CTEPH.

### sCD25 and NGAL correlate with markers of systemic and pulmonary inflammation

To probe if the leukocyte activation markers reflect systemic or pulmonary inflammation or are released from the pulmonary circulation we correlated levels with markers of systemic (CRP) and pulmonary (SpD) inflammation. As shown in Supplementary Table S1, NGAL and sCD25 consistently correlated positively with both CRP and SpD while sCD163 correlated with CRP only. Thus, the differences in sCD25 and sCD163 and to some degree NGAL levels between patients (including sub-groups) and controls were markedly attenuated when adjusting for CRP levels (Supplementary Figure S1). A similar effect for sCD25 and NGAL was seen with alleviated differences between the groups when including SpD in the adjustment.

Different levels of leukocyte activation markers in mixed venous blood from the pulmonary artery compared to arterial blood from the femoral artery could indicate local “production”/activation in the lungs. However, there were no significant difference in the levels of the markers between mixed venous blood (i.e. pulmonary artery) and arterial blood (i.e. femoral artery) (Table 2).

**Table 2:** Levels of soluble markers of leucocyte activation in plasma from the femoral (AF) and pulmonary (AP) artery in precapillary pulmonary hypertension at baseline, follow-up, and their change value during follow-up (median 4 months).

	Baseline	Follow-up	Change
**sCD25**			
AF	0.45 (0.26, 0.74)	0.66 (0.27, 0.85)*	0.04 (− 0.04, 0.37)
AP	0.66 (0.29, 0.96)	0.64 (0.32, 0.89)	0.04 (− 0.03 0.39)
**sCD163**			
AF	674 (493, 914)	651 (498, 854)	− 10 (− 261, 168)
AP	675 (544, 892)	687 (552, 922)	− 39 (− 193, 218)
**sCD14**			
AF	1.93 (1.69, 2.23)	1.99 (1.60, 2.30)	0.03 (− 0.10, 0.18)
AP	1.95 (1.69, 2.28)	2.09 (1.60, 2.31)	− 0.02 (− 0.14, 0.17)
**MPO**			
AF	150 (105, 187)	143 (100, 188)	− 7 (− 46, 47)
AP	155 (105, 205)	149 (104, 181)	− 7 (− 43, 39)
**NGAL**			
AF	64.9 (45.5, 91.5)	65.8 (46.2, 88.3)	4.02 (− 7.84, 17.9)
AP	59.0 (41.9, 91.0)	59.1 (46.0, 95.6)	5.16 (− 10.7, 19.1)

*p < 0.05 versus baseline, †*p* < 0.05 AF versus AP.

### Modest association between leucocyte markers, pulmonary pressures and cardiac function

In the patients with PH, we found modest associations between the markers of leucocyte activation and clinical and hemodynamic features. The findings were similar in arterial and mixed venous plasma (Supplementary Table S1). MPAP correlated negatively with sCD25, RAP correlated positively with sCD163, PaSO_2_ correlated negatively with sCD25 and sCD163, peakVO_2_ correlated negatively with sCD25, sCD14 and sCD163, eGFR correlated negatively with sCD25, sCD14 and NGAL, while NT-proBNP correlated positively with sCD25. Thus, although the correlations were rather modest, sCD163 showed an interesting pattern with a positive association with RAP and a negative association with PaSO2 without any associations with eGFR.

Evaluating the association in etiological subgroups revealed that the associations were stronger in the APAH group, in particular for sCD25 (Supplementary Table S2).

### Independent association of sCD163 with all-cause mortality in PH

**Figure 2:**
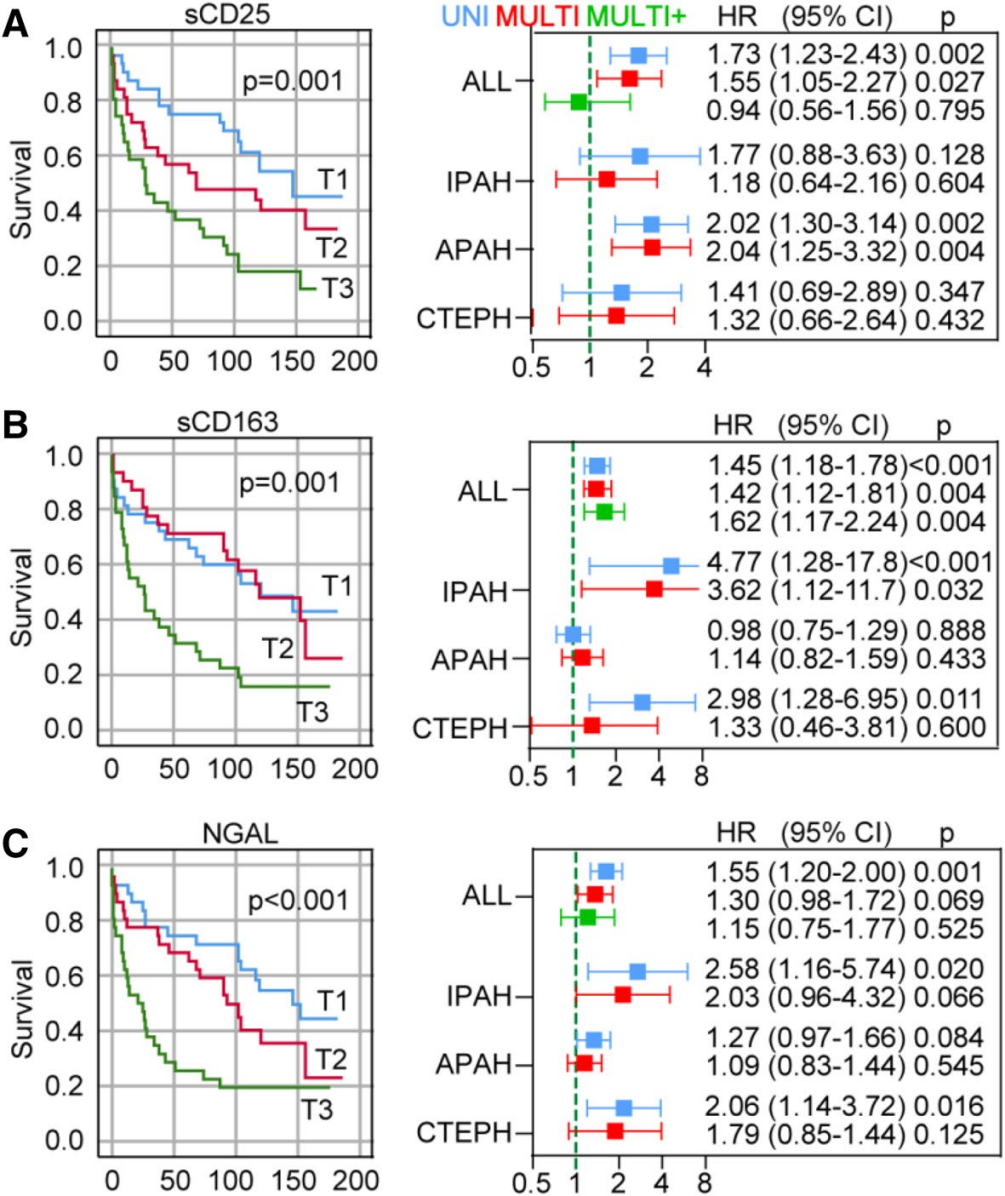
Soluble markers of leukocyte activation and all-cause mortality in precapillary pulmonary hypertension. (**A**) Left, Kaplan Meier curves showing associations between tertiles (ordered distribution into three parts, each containing a third of the cohort) (T1–T3) of sCD25 and all-cause mortality in plasma obtained from the pulmonary artery, *p*-values are from the log-rank test. Right, uni (blue) and multivariable (red, adjusting for age, sex, CI, RAP, PaSO_2_, eGFR, CRP, NT-proBNP; green, as for red + peak VO_2_) cox regression showing hazard ratios (HR), 95% CI and *p*-values for sCD25 in the whole patient population and according to etiology. IPAH, idiopathic pulmonary hypertension; APAH, acquired pulmonary hypertension; CTEPH, chronic thromboembolic pulmonary hypertension. (**B**) and (**C**), same as (**A**) but for sCD163 and NGAL, respectively.

During follow-up (median 90 months [range 0.5–193 months]) 67 of 106 patients died. As shown in the Kaplan Meier curves in Fig. 2, increasing pulmonary arterial plasma levels of sCD25, sCD163 and NGAL were associated with long-term, all-cause mortality. Results from multivariable cox regression are shown in the forest plot on the right side of Fig. 2 and complete survival data from femoral and pulmonary plasma are presented in Supplementary Table S3. After adjusting for age, sex and the strongest hemodynamic and biochemical predictors of long-term outcome (PaSO_2_, RAP, CI, eGFR, NT-pro-BNP and CRP as shown in Supplementary Table S3) sCD163 and sCD25 were still associated with all-cause mortality. The addition of peakVO_2_ to the model had little influence on the association between sCD163 and all-cause mortality, in contrast to sCD25 where the association with all-cause mortality was markedly attenuated and no longer associated with outcome. CRP was not associated with all-cause mortality in multivariable analysis (HR = 0.71, *p* = 0.17) and did not influence the association between sCD163 and all-cause mortality. When evaluating associations within different etiologies and using a propensity score (i.e., without including peakVO_2_) for adjustment, some patterns were revealed. As shown in Fig. 2, leukocyte markers in the pulmonary artery, sCD25 was associated with poor prognosis in APAH only, while sCD163 were associated (not significant) with poor prognosis in IPAH.

### Change in levels of leucocyte markers and all-cause mortality

Follow-up samples (median 4 months, range 1–31 months) were obtained in 49 (46%) of the patients. Of these, 28 died (57%) during a median of 108 months follow-up (range 4–186 months). As shown in Supplementary Table S4, there were no significant differences in the changes in leukocyte markers between survivors and non-survivors. In the population without a follow-up sample, 39 died (68%). Supplementary Table S5 details the difference in change in biochemical and hemodynamic features between survivors and non-survivors during 4 month follow-up. A smaller decline in MPAP, PVR and CRP and a smaller increase in CI were observed in non-survivors.

## Discussion

Our results demonstrate that (1) there is a systemic increase in soluble activation markers of different leucocyte subsets, in particular those reflecting monocyte and T-cell activation, in precapillary PH with mainly high levels in APAH (2) the monocyte/macrophage marker sCD163 was independently associated with poor long-term prognosis in the group as a whole (3) the T cell marker sCD25 was associated with poor prognosis in APAH, while sCD163 and the neutrophil marker NGAL were associated with poor prognosis in IPAH and CTEPH. Our data suggest that distinct patterns of leukocyte activation are associated with etiology and outcomes in precapillary PH. The association of sCD163, also after full adjustment, in the patient group as a whole is of particular interest.

The excessive vascular remodeling in pulmonary hypertension is associated with infiltration of inflammatory cells, including T cells and monocytes/macrophages. Our study suggests that this activation is reflected by markers of systemic activation showing higher levels of sCD163 and sCD25 with particularly high levels in patients with APAH. We observed no difference in level of any marker between plasma obtained from mixed venous blood from the pulmonary artery versus arterial blood from the femoral artery indicating that the markers do not reflect accumulation, release or activation in the pulmonary circulation. Furthermore, sCD25, sCD163 and NGAL, representing the markers most closely associated with poor prognosis, correlated with CRP as a measure of systemic inflammation indicating they partly reflect a general state of enhanced inflammation. However, while CRP itself gave prognostic information, CRP did not influence the association between leukocyte activation markers and poor prognosis, suggesting these markers do not merely reflect enhanced systemic inflammation. Indeed, both sCD25 and NGAL correlated positively with SpD as a measure of pulmonary inflammation^15^.

Elevated sCD163 has previously been reported in 26 patients with stable PAH^7^ and in systemic sclerosis, where it correlated with right-sided cardiac function and disease progression^16^. A major finding in the present study was that sCD163 was independently associated with poor outcome also after full adjustment of established hemodynamic markers, kidney function, NT-proBNP and peakVO_2_. In subanalysis, the prognostic value of sCD163 seemed to be restricted to IPAH. Since increased sCD163 levels are observed in autoimmune diseases driven by activated macrophages^17–19^ and autoimmune disease is a major component of APAH, the enhanced sCD163 levels in these patients may possible be secondary to their systemic disorders, and less implicated in progression of PH and poor prognosis. Moreover, perivascular infiltration of macrophages in pulmonary vascular lesions have also been observed in patients with IPAH^20^ and CTEPH^21^, and the stronger association with prognosis in these patients, may suggest that systemic levels to a larger degree reflect monocyte/macrophage activation in pulmonary tissues in IPAH in patients. Elevated sCD163 is released by proteolysis during active inflammation^22^, and it has also been suggested that sCD163 reflect the degree of activation of the pro-resolving and anti-inflammatory M2-like macrophages^23^. However, whereas enhanced release of pro-resolving mediators could be beneficial, enhance release of for example transforming growth factor-β, a major product of M2 macrophages, could promote PAH rather than resolve it^5^. In fact, it has been suggested that M2 macrophages predominate in the progression of PAH and may associate with pulmonary vascular remodeling^24^. Similar to sCD163, sCD14 was elevated in PAH but displayed no difference according to etiology. Ranchoux et al. also detected elevated sCD14 in PAH with no differences between iPAH and heritable PAH and linked the elevated levels to bacterial translocation and gut-lung interactions^25^. Possibly, this could explain the different association with regard to etiology between sCD163 and sCD14 as well as the different association with prognosis.

In contrast to sCD163 that was not associated with poor outcome in APAH, the T cell marker sCD25 was significantly associated with poor prognosis in this PH subgroup. Dysregulated T cell numbers and function has frequently been reported in all forms of PAH^20,26–30^, and contribute to the development and the progression of the disease. The present study, however, is to the best of our knowledge, the first report of raised levels of the general T-cell marker sCD25 in PH with particularly high levels in APAH. In relateion to the T-cells in PH, particular focus has been directed against abnormalities of regulatory T-cells (Treg) where increased systemic levels^27^, but reduced numbers in the lungs of PAH patients has been shown^20^. Deficient Treg cell activity in the lungs could blunt the response to inflammatory endothelial injury, including the pathogenic response of Th17 cells, thereby promoting PAH progression^30^. Since sCD25 reflects overall T-cell activation and not particular subsets, it is not known if elevated levels reflect high Th17/Treg ratios. However, sCD25 has been shown to directly enhance Th17 responses through its ability to sequester IL-2 and inhibit signaling downstream of IL-2R^31^ and could potentially be a mediator and not only a marker of T cell dysregulation in precapillary PH. Cause of death in APAH is most frequently due to end-stage HF and patients are characterized by low MaxVO2, which also completely mitigated the predictive ability of sCD25 when included in the cox regression model. We therefore speculate that the association between sCD25 and these indices, as well as NT-proBNP, may suggest a role for activated T cells in the transition to end-stage HF in APAH, as shown in experimental HF models^32,33^.

The circulating markers MPO and NGAL reflect neutrophil activation and were elevated in all etiologies with the highest levels in IPAH and APAH. Neutrophils produce a wide range of substances that could contribute to vascular remodeling and promote inflammation in PH^34^. Recently, MPO has been implicated in the pathophysiology of PH by demonstrating increased plasma MPO levels in two independent PAH cohorts and showing a mechanistic link between MPO and adverse pulmonary vascular function. Furthermore, MPO predicted adverse outcome although adjustment was restricted to NT-proBNP^35^. In contrast, we observed no association between MPO and poor prognosis, but found NGAL levels to predict all-cause mortality and similar to sCD163, these associations were stronger in IPAH and CTEPH. However, in addition to neutrophils, NGAL is also produced by injured nephron epithelia and is an emerging marker of acute kidney injury with prognostic impact in populations with cardio-renal syndromes^36^. Indeed, impaired renal function is a frequent co-morbidity in PAH and predictor of mortality^37^, linked to cardiac dysfunction rather than PH^38^.

Hemodynamic measures are used to monitor disease course in PH and non-invasive options are of interest for risk assessment and management of patients. However, we observed no difference in change of any leukocyte activation markers between survivors and non-survivors in our study during 4 months follow-up. Possibly, this time is too short for any changes in these leucocyte markers between survivors and non-survivors. Also, a substantial number of patients died without a follow-up sample and earlier sampling may have revealed different temporal course of the markers related to outcome. Changes in hemodynamic variables were not markedly different between survivors and non-survivors and sCD25 and sCD163 had only modest associations to these measures so we would not expect the markers to follow hemodynamic measures. A multimarker approach using a combination of markers has been proposed in predicting prognosis of PH while a number of single biomarkers have been evaluated reflecting different pathological pathways^8,39^. The lack of association between the leukocyte activation markers and more established indices of disease progression suggest they may reflect other important functions or mechanisms of the disease and could therefore potentially add value in a multimarker approach. The current state-of-art treatment in PAH seems not to target key features of pathogenesis. Understanding the role of leukocyte activation relevant to PAH pathophysiology may discover new therapeutic tools, but our study also highlights that etiology of PAH may have to be considered during manipulation of immune responses.

The present study has some limitations such as a relatively low number of healthy controls that were not matched with the PAH patients with relation to age and gender. However, the comparisons between patients and controls were adjusted for these variables. Inclusion of control population with increased systemic inflammation like scleroderma, would give more information on if the elevated marker levels reflect inflammation in general or if they do relate to PH. Moreover, the data from this single center study will have to be confirmed in additional PH cohort. We did not isolate circulating immune cells from these patients, and were unable to perform flow cytometry, which could give important additional information regarding activation of leucocyte subsets. Nonetheless, our study demonstrates a general leukocyte activation in precapillary PH with different profiles and impact on prognosis according to etiology. The prognostic impact of sCD163 also in the fully adjusted model should be of particular interest.

## Supplementary information

Supplementary Information

## Data Availability

All data generated or analysed during this study are included in this published article (and its Supplementary Information file).

## Abbreviations

APAH: Associated pulmonary arterial hypertension
CTEPH: Chronic thromboembolic pulmonary hypertension
IPAH: Idiopathic pulmonary arterial hypertension
PH: Pulmonary hypertension
