# Supplementary material for: Distinct patterns of soluble leukocyte activation markers are associated with etiology and outcomes in precapillary pulmonary hypertension
Source: Sci Rep. 2020 Oct 29;10:18540. doi: 10.1038/s41598-020-75654-w (PMC7596076; doi:10.1038/s41598-020-75654-w)

Supplementary File

Distinct Patterns of Soluble Leukocyte Activation Markers are Associated with Etiology and Outcomes in Precapillary Pulmonary Hypertension

Tove Lekva, Lars Gullestad, Kaspar Broch, Pål Aukrust, Arne K. Andreassen, Thor Ueland

**Supplementary Table 1.** Correlation between circulating markers of leucocyte activation and clinical and hemodynamic features in precapillary pulmonary hypertension

|  | sCD25 | | sCD163 | | sCD14 | | MPO | | NGAL | |
| --- | --- | --- | --- | --- | --- | --- | --- | --- | --- | --- |
|  | AF | AP | AF | AP | AF | AP | AF | AP | AF | AP |
| Sex | -0.03 | -0.076 | -0.04 | -0.03 | -0.21* | -0.13 | -0.05 | -0.03 | 0.02 | 0.12 |
| Age | 0.32** | 0.34** | 0.13 | 0.22* | 0.06 | 0.11 | -0.01 | -0.08 | 0.28** | 0.33** |
| RAP | 0.10 | 0.11 | 0.31** | 0.25* | 0.05 | 0.05 | 0.07 | 0.01 | 0.13 | 0.05 |
| MPAP | -0.27** | -0.21* | -0.04 | 0.03 | -0.14 | -0.23 | 0.09 | 0.03 | 0.02 | 0.09 |
| PCWP | -0.04 | -0.08 | -0.02 | 0.02 | -0.06 | -0.07 | 0.01 | -0.08 | -0.02 | 0.05 |
| PaSO_2_ | -0.20 | -0.28** | -0.25* | -0.21* | -0.06 | -0.14 | 0.02 | 0.01 | -0.13 | -0.12 |
| FaSO_2_ | -0.11 | -0.17 | 0.06 | 0.07 | -0.13 | -0.14 | <0.01 | -0.04 | -0.16 | -0.13 |
| CI | -0.06 | -0.15 | -0.09 | -0.10 | 0.00 | -0.04 | 0.03 | 0.02 | -0.01 | -0.06 |
| PVR | -0.16 | -0.07 | 0.03 | 0.05 | -0.09 | -0.13 | 0.02 | 0.01 | -0.04 | -0.01 |
| PeakVO_2_ | -0.22 | -0.29* | -0.23* | -0.07 | -0.12 | -0.09 | 0.13 | 0.13 | -0.12 | -0.02 |
| eGFR | -0.32** | -0.33** | -0.16 | -0.20 | -0.21* | -0.21* | -0.04 | -0.03 | -0.34** | -0.31** |
| NT-proBNP | 0.24* | 0.29** | 0.18 | 0.17 | 0.16 | 0.07 | 0.12 | 0.03 | 0.19 | 0.17 |
| hsCRP | 0.25* | 0.26* | 0.38*** | 0.38*** | 0.07 | 0.11 | 0.18 | 0.20* | 0.29** | 0.32** |
| SpD | 0.40*** | 0.36*** | 0.14 | 0.12 | 0.14 | 0.23* | 0.10 | 0.15 | 0.30** | 0.31** |

AF/AP, plasma from the femoral and pulmonary artery; Pa/SaO_2_, pulmonary and femoral arterial oxygen saturation; CI, cardiac index; MPAP, mean pulmonary artery pressure; NT-proBNP, N terminal pro-brain natriuretic peptide; eGFR, estimated glomerular filtration rate; PCWP, pulmonary capillary wedge pressure; RAP, right atrial pressure; PVR; pulmonary vascular resistance, Wood units; SpD, surfactant protein D *P<0.05, **P<0.01

**Supplementary Table 2.** Correlation circulating markers of leucocyte activation and clinical and hemodynamic features in the different etiologies of precapillary pulmonary hypertension

|  | sCD25 | | | sCD163 | | | NGAL | | |
| --- | --- | --- | --- | --- | --- | --- | --- | --- | --- |
|  | IPAH | APAH | CTEPH | IPAH | APAH | CTEPH | IPAH | APAH | CTEPH |
| Sex | 0.26 | -0.27 | -0.14 | 0.21 | -0.23 | 0.11 | 0.30 | 0.01 | 0.24 |
| Age | 0.32 | 0.33* | 0.41* | 0.36 | 0.18 | 0.46** | 0.48* | 0.28 | 0.38* |
| RAP | -0.05 | 0.38* | -0.05 | 0.39* | 0.42* | -0.03 | 0.04 | 0.28 | -0.37* |
| MPAP | -0.27 | -0.22 | 0.13 | 0.03 | -0.15 | 0.16 | 0.05 | 0.08 | 0.11 |
| PCWP | 0.08 | -0.26 | 0.08 | -0.05 | 0.06 | 0.12 | 0.12 | 0.21 | -0.15 |
| PaSO_2_ | 0.07 | -0.53** | -0.23 | 0.01 | -0.25 | -0.27 | 0.22 | -0.31 | -0.13 |
| FaSO_2_ | -0.03 | -0.21 | -0.08 | -0.04 | 0.24 | 0.06 | -0.15 | -0.35* | 0.14 |
| CI | -0.11 | -0.25 | -0.26 | 0.11 | -0.16 | -0.31 | 0.14 | -0.01 | -0.22 |
| PVR | -0.29 | -0.01 | 0.18 | -0.15 | -0.01 | 0.13 | -0.34 | -0.07 | 0.08 |
| PeakVO_2_ | -0.10 | -.42* | -0.26 | 0.41* | -0.18 | -0.29 | 0.20 | -0.21 | 0.07 |
| eGFR | -0.27 | -.46** | -0.29 | -0.28 | -0.32* | -0.22 | -0.50* | -0.28 | -0.19 |
| NT-proBNP | 0.14 | 0.46** | 0.23 | -0.14 | 0.19 | 0.33 | -0.10 | 0.28 | 0.07 |
| hsCRP | 0.02 | 0.35* | 0.42* | 0.27 | 0.27 | 0.57** | 0.08 | 0.42** | 0.23 |
| SpD | 0.36 | 0.38* | 0.20 | 0.36 | 0.10 | -0.01 | 0.28 | 0.38* | 0.16 |

Pa/SaO_2_, pulmonary and femoral arterial oxygen saturation; CI, cardiac index; MPAP, mean pulmonary artery pressure; NT-proBNP, N terminal pro-brain natriuretic peptide; eGFR, estimated glomerular filtration rate; PCWP, pulmonary capillary wedge pressure; RAP, right atrial pressure; PVR; pulmonary vascular resistance, Wood units; SpD, surfactant protein D *P<0.05, **P<0.01

**Supplementary Table 3.** Multivariable cox-regression models for circulating markers of leucocyte activation (log transformed and normalized) with step-wise adjustment for confounders and predictors of all-cause mortality in precapillary PH. Hazard ratios (HR) and 95%CI (confidence interval) and p-value are shown.

|  |  | Univariate  HR (95% CI) p-value | + Age. sex. CI. RAP. PaSO_2_. eGFR.  NT-proBNP. CRP  HR (95% CI) p-value | + PeakVO_2_  HR (95% CI) p-value |
| --- | --- | --- | --- | --- |
| sCD25 | AF | **1.61 (1.17-2.23) 0.004** | **1.58 (1.08-2.30) 0.018** | 0.92 (0.56-1.51) 0.746 |
|  | AP | **1.75 (1.23-2.49) 0.002** | **1.61 (1.10-2.37) 0.015** | 1.03 (0.61-1.71) 0.907 |
| sCD163 | AF | **1.49 (1.20-1.86)<0.001** | **1.45 (1.12-1.88) 0.004** | **1.83 (1.27-2.64) 0.001** |
|  | AP | **1.43 (1.16-1.77) 0.001** | **1.39 (1.11-1.76) 0.005** | **1.59 (1.17. 2.16) 0.003** |
| sCD14 | AF | **1.53 (1.12-2.09) 0.008** | **1.58 (1.09-2.31) 0.017** | 1.20 (0.76-1.87) 0.432 |
|  | AP | **1.36 (1.03-1.81) 0.033** | **1.48 (1.06-2.08) 0.023** | 1.14 (0.74-1.76) 0.561 |
| MPO | AF | 1.28 (0.90-1.83) 0.176 | 1.25 (0.85-1.84) 0.260 | 1.13 (0.68-1.86) 0.646 |
|  | AP | 1.26 (0.85-1.87) 0.249 | 1.29 (0.85-1.95) 0.227 | 1.40 (0.78-2.51) 0.257 |
| NGAL | AF | **1.64 (1.23-2.19) 0.001** | **1.42 (1.04-1.95) 0.029** | 1.36 (0.87-2.11) 0.174 |
|  | AP | **1.49 (1.14-1.94) 0.003** | 1.26 (0.95-1.67) 0.104 | 1.17 (0.76-1.79) 0.476 |

AF/AP. plasma from the femoral and pulmonary artery. CI. cardiac index; PaSO_2_. pulmonary artery oxygen saturation; RAP. right arterial pressure; eGFR. estimated glomerular filtration rate; NT-proBNP. N terminal pro-brain natriuretic peptide.

**Supplementary Table 4.** Levels of circulating markers of leucocyte activation in plasma from the femoral (AF) and pulmonary (AP) artery and in patients with precapillary pulmonary hypertension at baseline. follow-up at median 4 months. and their change in survivors and non-survivors during long term follow-up.

|  |  | Survivors | | | Non-Survivors | | | Change p-value |
| --- | --- | --- | --- | --- | --- | --- | --- | --- |
|  |  | Baseline | Follow-up | Change | Baseline | Follow-up | Change |  |
| sCD25 | AF | 0.43 (0.32. 0.74) | 0.66 (0.30. 0.83) | 0.03 (-0.14. 0.36) | 0.46 (0.18. 0.76) | 0.66 (0.23. 1.23) | 0.04 (-0.01. 0.42) | 0.353 |
|  | AP | 0.46 (0.30. 0.77) | 0.60 (0.33. 0.87) | 0.06 (-0.11. 0.34) | 0.70 (0.21. 0.97) | 0.74 (0.27. 1.23) | 0.04 (-0.01. 0.57) | 0.735 |
| sCD163 | AF | 536 (477. 800) | 548 (456. 730) | -4.75 (-158. 129) | 789 600. 996 | 726 (541. 968) | -94.3 (-371. 170) | 0.585 |
|  | AP | 630 (513. 861) | 631 (421. 737) | -54.7 (-203. 73.8) | 813 (576. 983) | 740 (567. 1019) | 40.5 (-191. 260) | 0.374 |
| sCD14 | AF | 1.91 (1.61. 2.13) | 1.92 (1.84. 2.24) | 0.03 (-0.02. 0.18) | 1.94 (1.73. 2.34) | 2.21 (1.40. 2.33) | 0.02 (-0.12. 0.16) | 0.739 |
|  | AP | 1.89 (1.59. 2.18) | 2.03 (1.59. 2.15) | -0.01 (-0.14. 0.08) | 1.97 (1.74. 2.35) | 2.17 (1.53. 2.43) | -0.03 (-0.13.0.20) | 0.269 |
| MPO | AF | 123 (102. 178) | 154 (105. 186) | 2 (-25. 54) | 164 (118. 247) | 140 (95. 191) | -13 (-75. 44) | 0.179 |
|  | AP | 134 (99. 171) | 137 (101. 187) | 1 (-37. 50) | 177 (123. 241) | 162 (119. 183) | -16 (-60. 38) | 0.312 |
| NGAL | AF | 54.9 (41.3. 77.2) | 51.7 (44.4. 70.3) | 0.30 (-7.84. 10.7) | 73.8 (48.6. 101.9) | 80.0 (46.9. 109.2) | 7.49 (-10.6. 25.8) | 0.295 |
|  | AP | 49.5 (37.8. 91.0) | 54.3 (42.1. 66.2) | -4.45 (-14.7. 17.1) | 66.3 (43.6. 90.7) | 66.6 (49.0. 104.3) | 7.04 (-1.50. 21.9) | 0.086 |

**Supplementary Table 5.** Comparison of change (Δ) in clinical. biochemical and hemodynamic features between survivors and non-survivors in precapillary pulmonary hypertension

|  | Comparison median Δ-values | |
| --- | --- | --- |
|  | Alive | Dead |
| RAP | -1 | -1 |
| MPAP | -11 | -5* |
| PCWP | 0 | 0 |
| PaSO_2_ | -1 | 0 |
| FaSO_2_ | 5 | 5 |
| CI | 0.4 | 0.3* |
| PVR | -5.5 | -2.8** |
| PeakVO_2_ | 4.6 | 3.4 |
| NT-proBNP | -97 | -37 |
| CRP | -1.2 | 0* |
| SpD | -79 | -32 |

Pa/SaO_2_. pulmonary and femoral arterial oxygen saturation; CI. cardiac index; CRP. C-reactive protein; eGFR. estimated glomerular filtration rate; MPAP. mean pulmonary artery pressure; NT-proBNP. N terminal pro-brain natriuretic peptide; PCWP. pulmonary capillary wedge pressure; RAP. right atrial pressure; PVR. pulmonary vascular resistance. Wood units; SpD. Surfactant protein D *P<0.05. **P<0.01

**Supplementary Figure 1**. Levels of soluble markers of leucocyte activation in precapillary PH adjusting for systemic (CRP) and pulmonary inflammation (SpD). Patients with PAH were classified as idiopathic PAH (IPAH. n=30), associated PAH (APAH. n=44), and chronic thromboembolic pulmonary hypertension (CTEPH. n=32) and in healthy controls (CTR. n=23). Data are given as estimated marginal means (ng/ml) adjusted for age. sex and either CRP (A) or SpD (B). P value represents the overall group effect. *p<0.05. **p<0. 01 ***p<0.001 vs controls. †p<0.05. ††p<0.001 between etiologies.


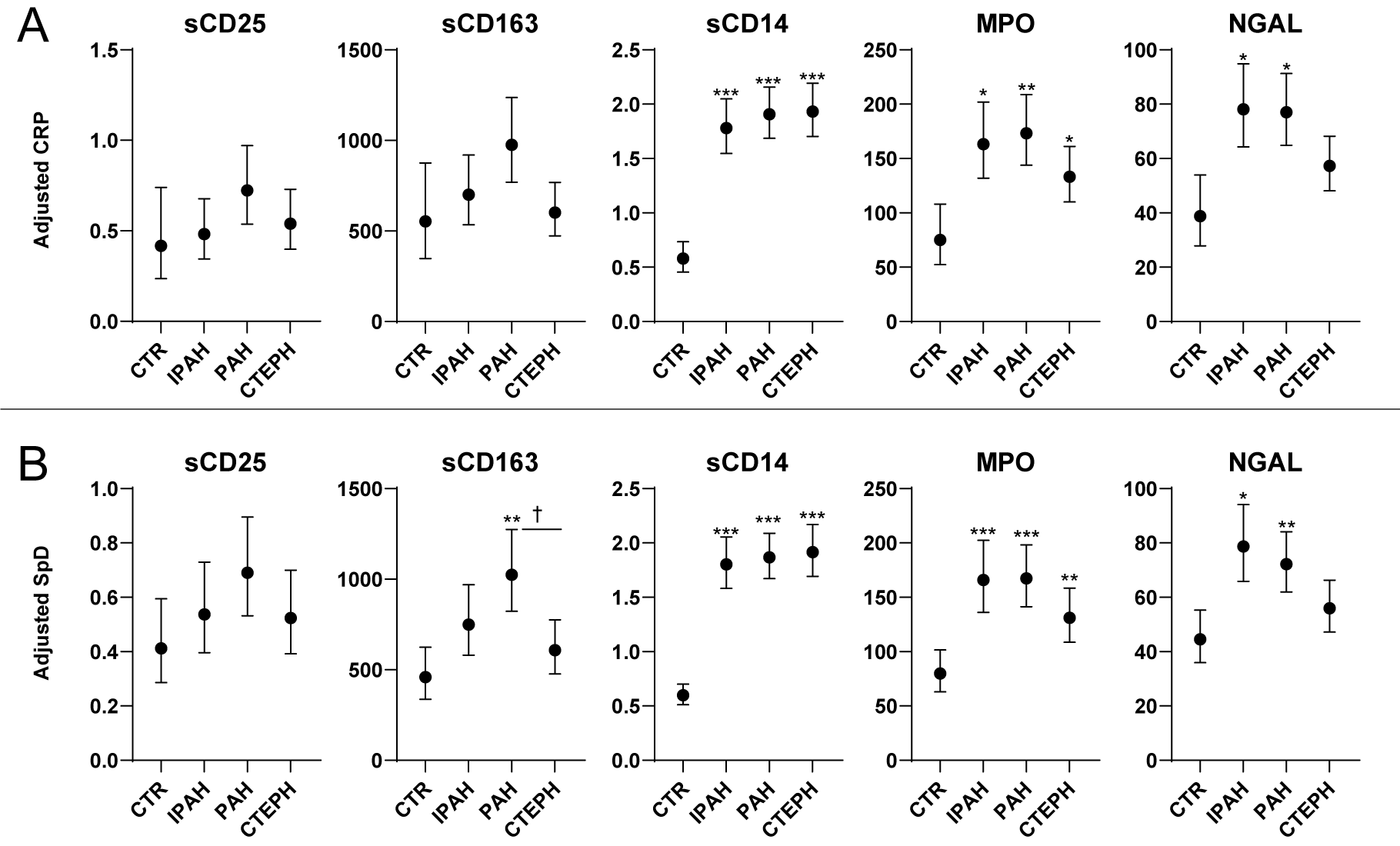


**Supplementary Figure 2**. Levels of soluble markers of leucocyte activation in precapillary PH from femoral artery. Patients with PAH were classified as idiopathic PAH (IPAH. n=30), associated PAH (APAH. n=44), and chronic thromboembolic pulmonary hypertension (CTEPH. n=32) and in healthy controls (CTR. n=23). Data are given as estimated marginal means (ng/ml) adjusted for age and sex. P value represents the overall group effect. *p<0.05. **p<0.01 ***p<0.001 vs controls. †p<0.05. ††p<0.001 between etiologies.

**
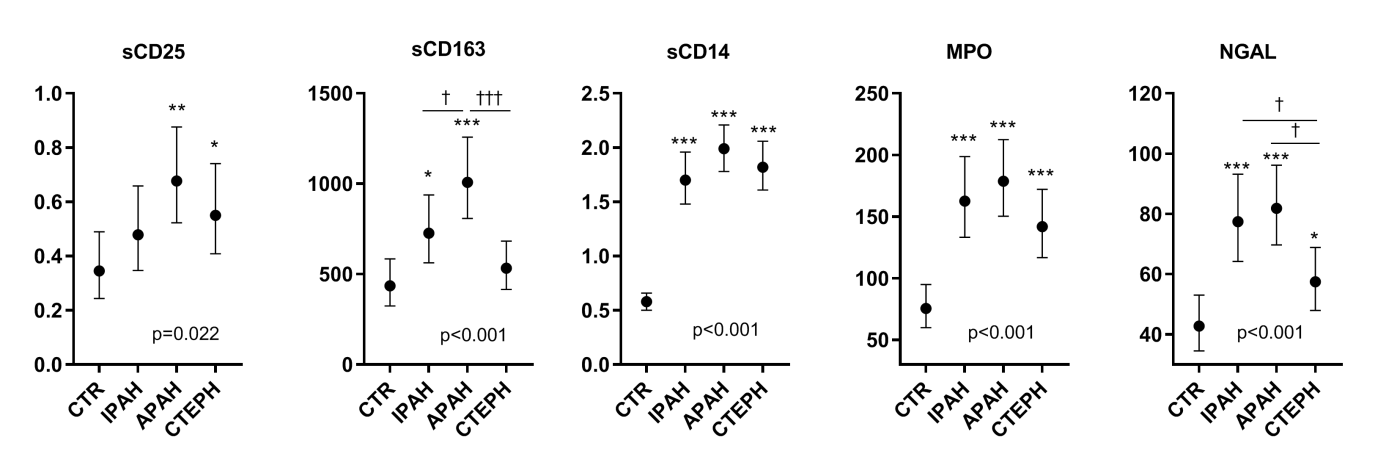
**

**Supplementary Figure 3**. Cox regression analysis showing univariate predictors of all-cause mortality. Fa/Pa-SO_2_ femoral and pulmonary arterial oxygen saturation; RAP; right arterial pressure. PVR. pulmonary vascular resistance; eGFR. estimated glomerular filtration rate; MPAP. mean pulmonary artery pressure. CI. cardiac index**.**


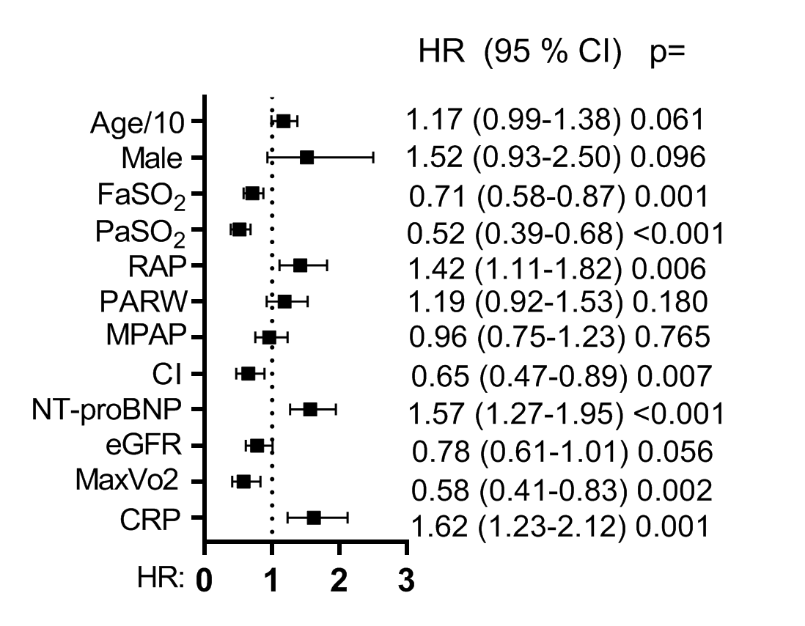

Supplement: Supplementary file 1 — Supplementary Information [file 41598_2020_75654_MOESM1_ESM.docx]
